# Primary Hepatic Leiomyosarcoma Presenting as a Multiloculated Cystic Mass: A Case Report and Review of Literature

**DOI:** 10.7759/cureus.99966

**Published:** 2025-12-23

**Authors:** Akhila Aravind, Navdeep Kaur, Youssef Al Hmada, Anita Choudhary, Satyapal Chahar

**Affiliations:** 1 Pathology, University of Mississippi Medical Center, Jackson, USA; 2 Pathology, Mayo Clinic, Rochester, USA; 3 Internal Medicine, St. Dominic Hospital, Jackson, USA; 4 Pathology/GI and Liver Pathology, University of Mississippi Medical Center, Jackson, USA

**Keywords:** hepatic abscess, hepatic leiomyosarcoma, mesenchymal, multiloculated cyst, primary hepatic leiomyosarcoma

## Abstract

Primary hepatic leiomyosarcoma (PHL) is a rare malignant mesenchymal tumor representing a small fraction of primary hepatic cancers. Due to its nonspecific clinical and radiologic presentation, it is often misdiagnosed. We present the case of an 82-year-old male who presented with altered mental status and was found to have a multiloculated cystic mass in the liver, initially suspected to be a hepatic abscess. Histopathological evaluation of the mass revealed a diagnosis of PHL. Despite palliative chemotherapy, the patient succumbed to metastatic disease within two months. PHL is a rare neoplasm with varied clinical manifestations. The unusual cystic presentation, as seen in this case, highlights the importance of maintaining a broad differential when evaluating hepatic masses.

## Introduction

Primary hepatic sarcomas are exceedingly rare, comprising only 0.2-2% of all primary malignant tumors of the liver, with leiomyosarcomas representing 6-16% of these sarcomas [1-3]. Primary hepatic leiomyosarcoma (PHL) is a malignant neoplasm of smooth muscle origin that may arise from intrahepatic blood vessels, bile duct musculature, or perisinusoidal myofibroblasts. Due to its rarity, PHL remains a poorly understood entity, with fewer than 100 cases reported in the literature to date [3,4].

Clinically, PHL lacks specific signs and symptoms, often manifesting with nonspecific complaints such as abdominal discomfort, weight loss, or constitutional symptoms. Imaging studies typically reveal a heterogeneous or cystic hepatic mass, often mimicking more common lesions such as hepatocellular carcinoma (HCC), intrahepatic cholangiocarcinoma, or pyogenic abscesses [1,4]. As a result, diagnosis is often delayed or missed until histopathologic evaluation is performed.

Immunohistochemistry plays a pivotal role in diagnosis, as the histologic features of PHL overlap with other spindle cell neoplasms of the liver. Tumor cells in PHL characteristically express smooth muscle markers such as SMA, desmin, and caldesmon, while being negative for markers of epithelial, neural, and gastrointestinal stromal differentiation [1,4,5]. Due to the high incidence of metastatic leiomyosarcomas to the liver from other primary sites, including the gastrointestinal tract, uterus, retroperitoneum, and lungs, exclusion of a non-hepatic primary is essential in confirming the diagnosis of PHL [2,4].

Herein, we present a case of PHL in an elderly male, initially suspected to be a hepatic abscess due to its multiloculated cystic appearance and clinical presentation. Through this case, we highlight the diagnostic challenges and clinical course of this rare malignancy, contributing to the limited body of literature on PHL.

## Case presentation

An 82-year-old male patient presented to the Emergency Department with acute confusion and altered mental status. His history was notable for a >20-pound weight loss over the past year. Physical examination and laboratory evaluation revealed a complicated urinary tract infection with urinary retention, hyponatremia, and *Escherichia coli* bacteremia. A renal ultrasound showed a markedly enlarged prostate (7.4 cm), warranting abdominal imaging to rule out a possible prostate abscess.

**Figure 1:**
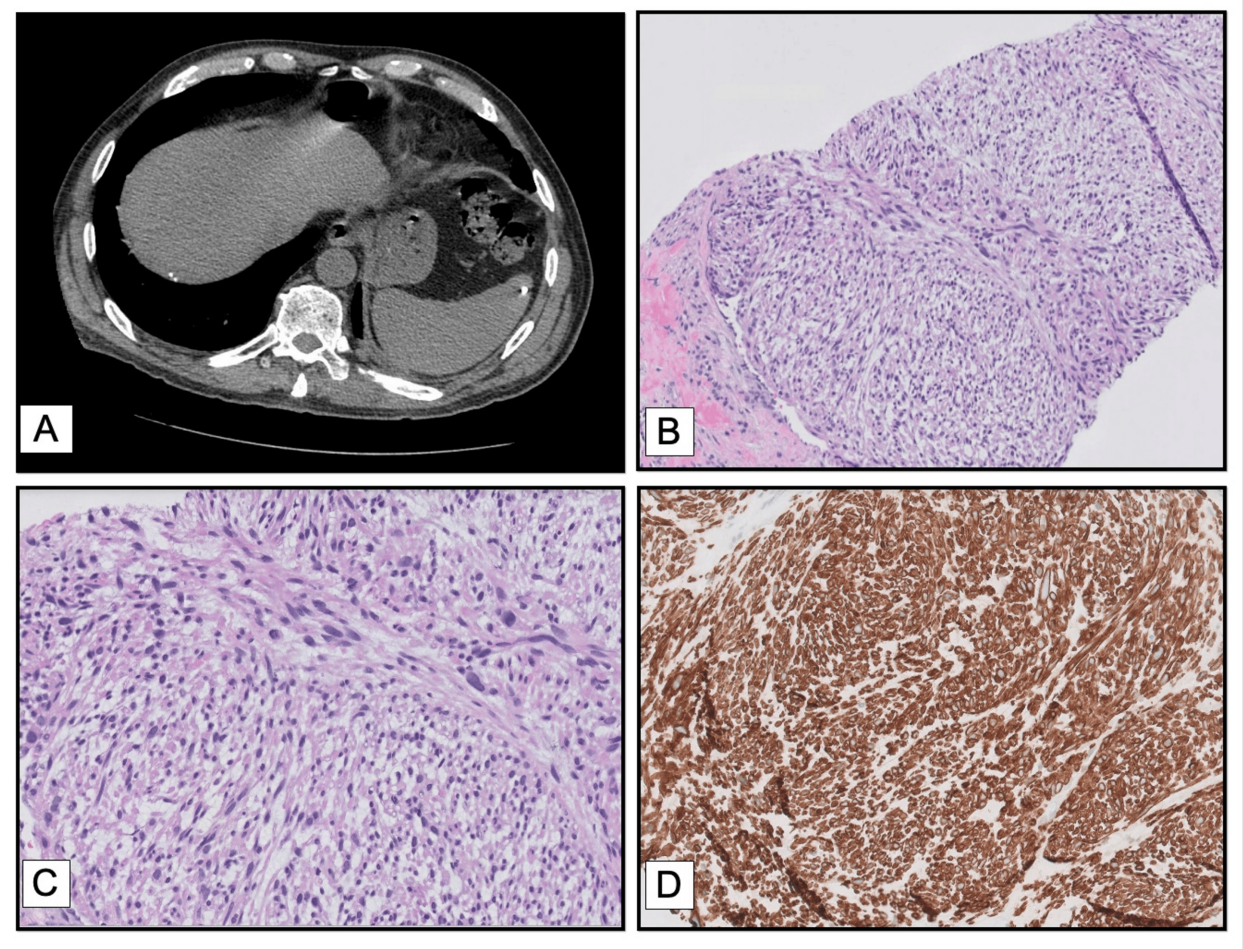
(A) CT Abdomen showing the multiloculated cystic mass; (B) H&E section from the biopsy (10 x); (C) H&E section from the biopsy (20x); (D) Immunohistochemistry stain – Caldesmon

Contrast-enhanced CT of the abdomen revealed a 6.6 × 4.2 × 3.7 cm multiloculated hypodense mass in the inferior right hepatic lobe with peripheral enhancement (Figure 1A). 

The lesion was initially suspected to be a hepatic abscess. The patient underwent image-guided aspiration and biopsy of the mass. Microbiologic cultures were negative. Histopathological examination demonstrated a spindle cell neoplasm with moderate nuclear atypia and elevated mitotic activity (Figure 1B, 1C). Immunohistochemical studies revealed tumor cell positivity for smooth muscle actin (SMA), desmin, and caldesmon (Figure 1D), with negative staining for cytokeratin, DOG1, and CD117. These findings were consistent with leiomyosarcoma.

Later, a whole-body PET scan was performed, which revealed metastases to the lungs, prostate, adrenal gland, and multiple lymph nodes. The case was discussed in the GI and hepatobiliary multidisciplinary tumor board, where it was determined that the patient was not a candidate for surgical resection. He was started on palliative chemotherapy but died two months following diagnosis.

## Discussion

Leiomyosarcomas are aggressive mesenchymal tumors derived from smooth muscle cells and constitute about 10-20% of all soft tissue sarcomas [2]. While most commonly located in the uterus and retroperitoneum, primary involvement of the liver is extremely rare. Among hepatic sarcomas, angiosarcoma remains the most frequent, followed by undifferentiated pleomorphic sarcoma and leiomyosarcoma [3,4].

PHL can present at any age, with reported cases ranging from infancy to the elderly. Although some reviews suggest a slight male predominance, there is no consistent sex distribution. The mean age at diagnosis is around 51 years, but cases have been documented in patients as old as 86 years [3,4]. The etiology remains elusive, although associations have been made with immunosuppression (notably HIV/AIDS), Epstein-Barr virus (EBV) infection, post-organ transplantation, hepatitis C virus (HCV)-related cirrhosis, thorotrast exposure, and prior radiation or chemotherapy for other malignancies such as Hodgkin lymphoma [5-9].

The clinical presentation of PHL is often insidious and nonspecific, including symptoms such as abdominal pain, fever, weight loss, and fatigue. In rare cases, the tumor may mimic a hepatic abscess, as observed in our patient, leading to diagnostic confusion [1]. Radiologically, PHL may appear as a well-demarcated mass with peripheral enhancement and cystic or necrotic areas, but these findings are not pathognomonic. Therefore, a definitive diagnosis relies on tissue biopsy and immunohistochemical analysis [1,3]. Our case also presented a similar scenario, where the clinical features were misleading, but tissue biopsy played a crucial role in establishing an accurate diagnosis.

Histologically, PHL shows intersecting fascicles of spindle-shaped cells with eosinophilic cytoplasm, nuclear atypia, and high mitotic activity. Immunohistochemically, these tumors are positive for smooth muscle markers such as SMA, desmin, and caldesmon, and negative for epithelial (cytokeratins), gastrointestinal stromal (DOG1, CD117), and neural (S-100) markers [1,4]. This immunoprofile helps differentiate PHL from gastrointestinal stromal tumors (GISTs), inflammatory myofibroblastic tumors (IMTs), sarcomatoid hepatocellular carcinoma, and other spindle cell neoplasms of the liver. It is important to clearly distinguish PHL from other similar tumors, not just for diagnosis but also because the treatment plans are different for each of them [2,3,10].

Surgical removal of the tumor with clear margins (R0 resection) continues to be the main and only potentially curative treatment for PHL. Complete surgical excision offers the best chance for long-term survival, as these tumors generally do not respond well to chemotherapy or radiotherapy. Unfortunately, because the symptoms are vague and usually appear late, most patients, including ours, are diagnosed at an advanced stage or already have metastatic disease, which makes curative surgery difficult or not possible. As reported by Esposito et al., patients who underwent complete (R0) resection had a median survival of 37.5 months and a five-year disease-specific survival rate of about 67% [3]. On the other hand, patients with inoperable or metastatic disease tend to do much worse, with median survival often limited to a few months to one year, depending on the tumor spread and how well they respond to palliative treatment. Therefore, early diagnosis and the possibility of surgical removal are key factors in improving outcomes in PHL.

Currently, no standardized treatment protocols exist for unresectable PHL. Systemic chemotherapy regimens used for soft tissue sarcomas, such as doxorubicin and ifosfamide, are commonly used, though their efficacy in PHL remains uncertain [2,4]. Our patient received palliative chemotherapy following multidisciplinary consensus but succumbed to disease within two months, consistent with the aggressive nature and poor outcomes associated with metastatic PHL.

This case emphasizes the importance of considering rare differential diagnoses when encountering atypical hepatic masses, especially in patients without clear risk factors for common hepatic malignancies. Awareness of this entity and its mimics is essential for timely diagnosis and management.

## Conclusions

PHL is a rare malignant tumor often diagnosed at an advanced stage due to its non-specific presentation and is associated with a poor prognosis. Most hepatic leiomyosarcomas are metastatic, commonly originating from the gastrointestinal tract, uterus, retroperitoneum, or lung. Thus, excluding metastatic disease is essential when diagnosing PHL. Its occurrence as a multiloculated cystic lesion mimicking a hepatic abscess is particularly uncommon, underscoring the need for histological confirmation in atypical hepatic masses.
